# Lack of Molecular Mimicry between Nonhuman Primates and Infectious Pathogens: The Possible Genetic Bases

**DOI:** 10.1055/s-0041-1724106

**Published:** 2021-02-19

**Authors:** Darja Kanduc

**Affiliations:** 1 Department of Biosciences, Biotechnologies, and Biopharmaceutics, University of Bari, Bari, Italy

**Keywords:** infectious agents, peptide sharing, molecular mimicry, cross-reactivity, autoimmunity, nonhuman primates, rhesus macaque, vaccines, preclinical test

## Abstract

Recently, it was found that proteomes from poliovirus, measles virus, dengue virus, and severe acute respiratory syndrome-related Coronavirus 2 (SARS-CoV-2) have high molecular mimicry at the heptapeptide level with the human proteome, while heptapeptide commonality is minimal or absent with proteomes from nonhuman primates, that is, gorilla, chimpanzee, and rhesus macaque. To acquire more data on the issue, analyses here have been expanded to Ebola virus, *Francisella tularensis* , human immunodeficiency virus-1 (HIV-1), *Toxoplasma gondii* , Variola virus, and *Yersinia pestis* . Results confirm that heptapeptide overlap is high between pathogens and *Homo sapiens* , but not between pathogens and primates. Data are discussed in light of the possible genetic bases that differently model primate phenomes, thus possibly underlying the zero/low level of molecular mimicry between infectious agents and primates. Notably, this study might help address preclinical vaccine tests that currently utilize primates as animal models, since autoimmune cross-reactions and the consequent adverse events cannot occur *in absentia* of shared sequences.

## Introduction

Beginning in 2000, a high, unexpected level of molecular mimicry between microbial and human proteins has been repeatedly documented; accordingly, the consequent potential cross-reactivity following infections or active immunizations has been highlighted. 1 2 3 4 5 6 7 8 9 10 11 Pathologically, cross-reactions between pathogen and human proteins might lead to thrombocytopenia, altered spermatogenesis, schizophrenia and neuropsychiatric diseases, neurodegeneration, lymphomas, sudden death, microcephaly and Guillain-Barré syndrome, pneumonia, multiple sclerosis, immunodeficiency, developmental disorders, autoinflammatory disease, arthritis, hemochromatosis, myasthenia gravis, and systemic lupus erythematosus. 4 8 12 13 14 15 16 17 18 19 20 21 22 23 24 25 26

As a matter of fact, pathogen-derived immunoreactive epitopes are mostly composed of peptide sequences present in human proteins, 10 18 21 23 26 thus documenting that the immune system does not exert any negative selection of self-reactive lymphocytes. 27 28 Hence, it comes as a logical consequence that peptide sharing between infectious antigens and human proteins can cause cross-reactions in the human host, possibly leading to a multitude of postinfection autoimmune pathologies.

However, as recently underlined, 29 30 in general cross-reactivity and the related potential autoimmune sequelae have not been reported in nonhuman primates following experimental infections or during preclinical trials for vaccine validation. 31 Indeed, preclinical trial reports routinely state that active antipathogen immunization is exempt from adverse events in the animal model *par excellence,* namely, rhesus macaque ( *Macaca mulatta* ), which is a nonhuman primate phylogenetically close to humans. 32 33 34 35 36

Therefore, it was hypothesized that if the peptide sharing between pathogens and humans is the *primum movens* of autoimmune pathologies via cross-reactivity, then different levels of peptide sharing with pathogens should characterize the proteomes of humans and primates. *De facto* , comparative amino acid (aa) sequence analyses documented that poliovirus, measles virus, dengue virus, and SARS-CoV-2 share peptide sequences almost exclusively with the human and murine proteomes, but not with primate proteomes. 29 30 These data might explain the absence of collateral adverse events in primates during preclinical vaccine tests, since autoimmune cross-reactivity cannot occur in primates *in absentia* of molecular mimicry.

In this research frame, the present study expands comparative sequence analyses to additional pathogens that, although thoroughly investigated, still remain without safe and efficacious therapies, for example, *Yersinia pestis* 37 and HIV-1. 38 The results further suggest that primates are animal models unsuitable to show adverse autoimmune pathologic cross-reactions in pre-clinical trials following pathogen administration by infection or active immunization, and indicate that only mice represent animal models suitable to test putative vaccine candidates. The genetic bases that might underlie the low level of heptapeptide sharing between infectious agents and nonhuman primates are discussed.

## Materials and Methods

Molecular mimicry analyses were conducted by using heptapeptides as immunobiological units. The analyzed pathogen proteins/proteomes are as follows (with NCBI TaxId in parentheses and further details at http://www.ncbi.nlm.nih.gov/Taxonomy/Browser/wwwtax.cgi ): membrane protein/O-antigen protein from *Francisella tularensis* , 409 aa (177416); apical membrane antigen 1-like protein from *Toxoplasma gondii* , 651 aa (432359); surface antigen S from variola virus, 354 aa (587200); virulence-associated V antigen from *Yersinia pestis* , 326 aa (632); proteome from Ebola virus, 5494 aa (128952); proteome from HIV-1, 3134 aa (11676).

The primary aa sequences of the pathogen proteins/proteomes were dissected into heptapeptides offset by one residue, that is, MIRAYEQ, IRAYEQN, RAYEQNP, and so on. Then, each pathogen heptapeptide was analyzed for occurrences within mammalian reference proteomes, that is, proteomes that have been selected because they cover well-studied model organisms and other organisms of interest for biomedical research and phylogeny ( www.uniprot.org/proteomes ). 39 40 41 Specifically, analyses were conducted on proteomes from the following organisms (with NCBI TaxId in parentheses): *Homo sapiens* (9606); gorilla, *Gorilla gorilla gorilla* (9595); chimpanzee, *Pan troglodytes* (9598); and rhesus macaque, *Macaca mulatta* (9544). In addition, proteomes from the following mammalian organisms were analyzed as controls: cow, *Bos taurus* (9913); dog, *Canis lupus familiaris* (9615); cat, *Felis catus* (9685); rabbit, *Oryctolagus cuniculus* (9986); mouse, *Mus musculus* (10090); rat, *Rattus norvegicus* (10116); pig, *Sus scrofa* (9823); and bat, *Pteropus alecto* (9402).

Heptapeptide matches between pathogen proteins/proteomes and mammalian proteomes were searched using Pir Peptide Match program (research.bioinformatics.udel.edu/peptidematch) 40 and UniProt/Swiss-Prot database that is available at www.uniprot.org 39 and consist of reviewed and annotated protein entries. Protein isoforms were not considered.

## Results

**Fig. 1:**
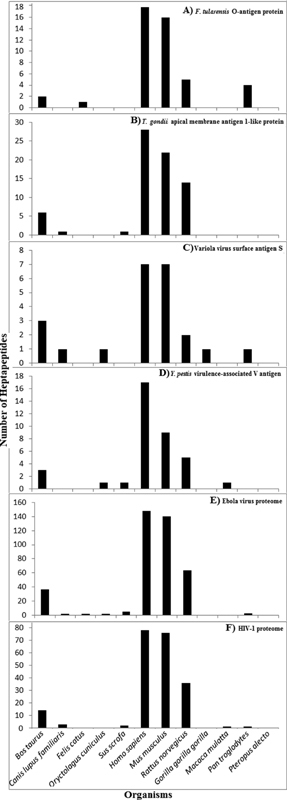
Heptapeptide sharing between mammalian proteomes and: ( **A** ) *F. tularensis* membrane protein/O-antigen protein, ( **B** ) *T. gondii* apical membrane antigen 1-like protein, ( **C** ) variola virus surface antigen S, ( **D** ) *Y. pestis* virulence-associated V antigen, ( **E** ) Ebola virus proteome, and ( **F** ) human immunodeficiency virus (HIV)-1 proteome.

We analyzed four protein antigens derived from *F. tularensis* , *T, gondii* , variola virus, and *Y. pestis* , respectively, and two pathogen proteomes, namely, Ebola virus proteome, and HIV-1 proteome for heptapeptide sharing with the mammalian proteomes described under Methods. The heptapeptide sharing is quantitatively reported in Fig. 1 and qualitatively illustrated in Supplementary Tables S1–S6 (online only).

As a preliminary observation, it is noteworthy, as already underscored elsewhere, 8 9 10 that the peptide sharing shown in Fig. 1 is highly improbable from a mathematical point of view. Indeed, the expected number of times that one heptapeptide from a protein will occur simultaneously in a second protein is given by the formula *mn* /N, where *m* is the number of heptapeptides present in the first protein, *n* is the number of heptapeptides present in the second protein, and N is 20 7 = 1,280,000,000, that is, the number of heptapeptides that can be composed using the 20 aa. For values of *m* and *n* << N, the probability of sharing only one heptapeptide is 0.00000000078125, that is infinitesimal.

Then, Fig. 1 shows that the peptide sharing is not stochastic, that is, the pathogen-derived heptapeptides are not distributed at random among the analyzed mammalian proteomes. Indeed, all of the analyzed pathogen proteins/proteomes, independently of their being bacterial or viral or protozoan, and independently of their aa length, share heptapeptide sequences almost exclusively with the human, murine, and rat proteomes. Zero or a low number of pathogen-derived heptapeptides are present in the proteomes from cat, dog, cow, pig, rabbit, and the three primates. As a logical consequence, Fig. 1 shows that pathologic cross-reactivity following pathogen infection/immunization might be revealed only by using mice as animal models in preclinical tests.

On the whole, the data exposed in Fig. 1 might explain the differences between humans and primates in the incidence or severity of medical conditions. In fact, communicable and noncommunicable diseases that are common in humans are practically absent or very rare in great apes. 42 43 44 Taking HIV-1 infection as an example, the progression to AIDS—common in humans and rare in great apes 44 —can be explained by the vast peptide sharing between HIV-1 and human proteins that—when altered, mutated, deficient or improperly functioning—associate with AIDS disorders, that is, immunosuppression, neurological disturbances, muscle diseases, malignancies, lipodystrophies, diarrhea, bone loss, corneal alterations, kidney disease, and hypertension, among others, which most possibly associate with molecular mimicry. 45 Instead, cross-reactions and autoimmune pathologies cannot occur in animals that do not share peptides with HIV-1. In these animals, HIV-1 infection/active immunization will be well tolerated with no adverse events as routinely reported in preclinical tests conducted in Rhesus macaques. 46

Thus, Fig. 1F offers a scientific explanation of the numerous HIV vaccine failures. Indeed, as early as 2009, Thomas 47 wrote “ *to say that efficacy trials of HIV vaccines and microbicides have, to date, been disappointing is something of an understatement* .” Today, in 2020, Thomas' observation is still valid and the promised effective vaccine that had to cure HIV has not yet been found, 48 and most possibly will not be found within the next decades if correct trials and correct animal models are not adopted.

## Discussion

Recently, it was shown that heptapeptides from poliovirus, measles virus, dengue virus, and SARS-CoV-2 occur in the human proteome, but not in proteomes from primates and domestic animals. 29 30 The data appeared to be of relevance, since they might indicate that, starting from the very beginning of the mass polio vaccination program in 1962 49 until the current project of mass vaccination against SARS-CoV-2, 35 36 the human population has been vaccinated and revaccinated,, and it is intended to be vaccinated on the basis of protocols that used and use animal models unable to highlight adverse autoimmune pathologic consequences.

The present study confirms such previous data 29 30 and documents that a mathematically unexpected high molecular mimicry at the heptapeptide level occurs between high-risk pathogens, namely, *F. tularensis* , *T. gondii* , variola virus, *Y. pestis* , Ebola virus, and HIV-1, and the human proteome. Such high molecular mimicry is practically absent when proteomes from primates, domestic animals, and cattle are analyzed. Hence, this study might explain not only the wave of autoimmune diseases that are increasingly burdening the human population worldwide, 50 but also the repeated failures in defining immunotherapies for infectious diseases which pose a risk to public health and primary health care. 51 52 53 54 55 56 57 58 59 60

Also, it has to be considered that the present data underestimate the cross-reactivity potential by two orders of magnitude. Indeed, if one considers that a minimal immune determinant corresponds to five aa residues, 61 62 the extent of the peptide overlap of microbial versus human proteins and the consequent potential cross-reactivity risk increase exponentially. Moreover, conformational epitopes have not been considered.

In light of these additional caveats and of the consequent higher cross-reactivity risk, it appears to be mandatory to investigate the molecular mechanisms that underlie the different extents of molecular mimicry between pathogens and mammals. Possible objects of investigation might be, for example, alterations of gene transcription/translation potentially involved in the different shaping of human and primate genomes/phenomes.

In this regard, studies by Puente et al 63 already highlighted important differences in the human and chimpanzee genomes, from deletion of whole genes to small insertion/deletion events or single nucleotide changes that lead to specific gene inactivation. For example, the genes encoding Serine protease 33 (PRSS33/EOS) and Glutathione hydrolase 5 proenzyme (GGTLA1) are absent in chimpanzee, and single nucleotide changes in protease genes such as Inactive caspase-12 (CASP12) lead to functional genes in chimpanzee and pseudogenes in human. 63 Actually, although the nucleotide difference between humans and chimpanzees is surprisingly small with a value suggested to be 1 to 2%, it was reported that 80 percent of proteins are different between humans and chimpanzees. 64

In addition, segmental duplications in the genome and transposable elements are important sources of genetic/phenetic differences between humans and primates. Segmental duplications are blocks of highly homologous duplicated sequences that define hotspots of chromosomal rearrangement and act as mediators of normal variation as well as genomic diseases. 65 Studies of gene family evolution indicate that gene loss and gain are enriched within the primate lineage 66 67 and that recurrent and independent gene-containing duplications occur within the gorilla and chimpanzee, and are absent in the human lineage. 68 In particular, Blekhman et al 69 showed that not only species-specific segmental duplications are enriched with genes that are differentially expressed between species but, in addition, genes that are within species-specific segmental duplications show significantly higher absolute fold difference in expression level between human and chimpanzee compared with genes that are not associated with duplications. In this regard, it is worth mentioning that a large fraction of the KRAB-containing zinc finger (KRAB-ZF) genes—that code the largest family of transcription factors (TFs) in humans—arose from segmental duplications. 70 In primates, KRAB-ZF genes duplicate at a high rate. Due to their function as transcriptional repressors, the generation and rapid divergence of these genes may help to explain some of the transcriptome differences that have been documented between humans and our closest relatives among the apes. 71 72 73

In sum, it is not surprising that primates are not good models for many major human diseases/conditions 42 43 44 and for preclinical vaccine tests. 29 30 Literature data and the present data might explain the inefficacy and the problematics of vaccines, 51 52 53 54 55 56 57 58 59 60 thus inviting researchers and vaccinologists to study, identify, and use the correct animal models capable of revealing potential autoimmune pathogenicity connected to the peptide sharing.

Finally, as a conclusive note, it appears pertinent to recall the basic concept first stated in 2000 1 and then repeatedly illustrated ( 1 2 3 4 5 6 7 8 9 10 11 27 28 74 75 76 77 78 and additional references therein), according to which only pathogen-derived peptides, which are absent in the human proteome, that is, “non-self” peptides, can lead to safe and efficacious immunotherapies.
