# Supplementary material for: Lack of Molecular Mimicry between Nonhuman Primates and Infectious Pathogens: The Possible Genetic Bases
Source: Glob Med Genet. 2021 Feb 19;8(1):32–7. doi: 10.1055/s-0041-1724106 (PMC7964256; doi:10.1055/s-0041-1724106)
Supplement: Supplementary file 1 — Supplementary Material [file 10-1055-s-0041-1724106-s2100005.pdf]

Supplementary appendix: description of the heptapeptide sharing between pathogen proteins/proteomes and mammalian proteomes.

Supplementary Table S1 *Francisella tularensis* membrane protein/O-antigen protein, 409 aa (NCBI: TaxId 177416)

Organism	N°	Shared heptapeptides
Bos taurus	2	KEKLLFI, LFVGNIV
Canis lupus familiaris	0	–
Felis catus	1	YRSLFLL
Oryctolagus cuniculus	0	–
Sus scrofa	0	–
Homo sapiens	18	DGLGFSI, EKLLFII, FLLLLIV, FVGNIVK, GLGFSIT, IFISRTS, ILFIFSL, LFIIILA, LFLLLLI, LFVGNIV, LLFIIIL, LLLIVCL, LSNAGGD, LTSSLIL, NIFISRT, SNIFISR, TSLLTSS, TSSLILL
Mus musculus	16	DGLGFSI, EKLLFII, GLGFSIT, IFISRTS, IFSLKEN, ILFIFSL, LFLLLLI, LFVGNIV, LLLIVCL, LSNAGGD, LYLLAFC, NIFISRT, SIFVFLS, SLFLLLL, SNIFISR, TSLLTSS
Rattus norvegicus	5	DGLGFSI, GLGFSIT, IFISRTS, NIFISRT, SNIFISR
Gorilla gorilla gorilla	0	–
Macaca mulatta	0	–
Pan troglodytes	4	FLLLLIV, FVGNIVK, LFIIILA, LLFIIIL
Pteropus alecto	0	–

Supplementary Table S2 *Toxoplasma gondii* apical membrane antigen 1-like protein, 651 aa (NCBI: TaxId 432359)

Organism	N°	Shared heptapeptides
Bos taurus	6	HLSRLLR, LATPELQ, LPPTERP, NPSTNYT, VFGKAIQ, VFLLCDV
Canis lupus familiaris	1	LSRLLRA
Felis catus	0	–
Oryctolagus cuniculus	0	–
Sus scrofa	1	GEGGGGG
Homo sapiens	28	AGLVFLL, ALIAGSV, ASKKPLP, AVPPDHS, EKEGSGG, EPSVPEP, FLLCDVL, GEGGGGG, GGGGGTS, GGGTSGD, IAGSVLG, IAVNPST, KEGSGGN, LATPELQ, LLQERLG, LPPTERP, LPPTPGE, LSRLLRA, NPSTNYT, PGEGGGG, PLPPTER, PPTPGEG, PTEPSVP, PVFGKAI, RLLRAGL, SVNGTPS, TALIAGS, TESLLGS
Mus musculus	22	ASGGREV, ASKKPLP, EKEGSGG, ELQNTVI, EPPVVLP, GEGGGGG, GGGGGTS, IAGSVLG, LLFSSRS, LLQERLG, LSRLLRA, NPSTNYT, PGEGGGG, PIDDSLL, PSDVAVP, PSVPEPE, PVFGKAI, PVKEKSS, RLLRAGL, SLLQERL, SVNGTPS, TVEASGG
Rattus norvegicus	14	AGSVLGM, DAVSRKC, EPPVVLP, GEGGGGG, GGGGGTS, GGGGTSG, IAGSVLG, LIAGSVL, LLFSSRS, RLLRAGL, TVIRSSK, VFGKAIQ, VIRSSKA, YDAVSRK
Gorilla gorilla gorilla	0	–
Macaca mulatta	0	–
Pan troglodytes	0	–
Pteropus alecto	0	–

 © 2021. The Author(s).

Supplementary Table S3 Variola virus surface antigen S, 354 aa (NCBI: TaxId 587200)

Organism	N°	Shared heptapeptides
Bos taurus	3	LLLLFHS, LLLLLFH, SLLLLLF
Canis lupus familiaris	1	SLLLLLF
Felis catus	0	–
Oryctolagus cuniculus	1	SLLLLLF
Sus scrofa	0	–
Homo sapiens	7	AKDSKWL, DSGRYDC, EDSLLSR, ITWYKDN, IYFVSLL, SLLLLLF, VSLLLLL
Mus musculus	7	AKDSKWL, DSGRYDC, LEDSGRY, NNMAAIG, SLLLLLF, VSLLLLL, VSTSLLF
Rattus norvegicus	2	SLLLLLF, VSLLLLL
Gorilla gorilla gorilla	1	VSLLLLL
Macaca mulatta	0	–
Pan troglodytes	1	VSLLLLL
Pteropus alecto	0	–

Supplementary Table S4 *Yersinia pestis* virulence-associated V antigen, 326 aa (NCBI: TaxId 632)

Organism	N°	Shared heptapeptides
Bos taurus	3	DGSEKKI, NNELSHF, SVLEELV
Canis lupus familiaris	0	–
Felis catus	0	–
Oryctolagus cuniculus	1	NNELSHF
Sus scrofa	1	SSVLEEL
Homo sapiens	17	DDIELLK, DGSEKKI, DPRKDSE, ELTAELK, ESSPNTQ, IEDLEKV, KLREELA, LAYFLPE, LPEDAIL, NKHLSSS, NNELSHF, NSYSYNK, REELAEL, SKLREEL, SSVLEEL, SVLEELV, SVMQRLL
Mus musculus	9	DGSEKKI, DPRKDSE, IEDLEKV, LAYFLPE, LPEDAIL, NNELSHF, QLVKDKN, REELAEL, SVMQRLL
Rattus norvegicus	5	LPEDAIL, NNELSHF, QLVKDKN, REELAEL, SVLEELV
Gorilla gorilla gorilla	0	–
Macaca mulatta	1	SSVLEEL
Pan troglodytes	0	–
Pteropus alecto	0	–

Supplementary Table S5 Ebola virus proteome, 5494 aa (NCBI: TaxId 128952)

Organism	N°	Shared heptapeptides
Bos taurus	37	DGIRGFP, DLCNFLV, GILQLPR, IEDSKLR, KLRALLT, QQEEGPK, RGRPRAA, SKLRALL, AFHQLVQ, DGIRGFP, EEVVQTL, EVVQTLA, GGNSNTG, RGGNSNT, SLRPVPP, VNVISGP, DGIRGFP, GILQLPR, IIAVIAL, LTTKPGP, MASENSS, SGQSPAR, LTGLLSL, VLKVFLS, AAGDRGL, DPVTSGL, ELASRLT, PLKGGLN, FKEAVQG, GEHATVR, LGGLSFL, LLSILGS, EAEKQLQ, GAVKYLE, PDSLEEE, QLREAAT, VKRLEEL
Canis lupus familiaris	2	SKLRALL, GAVKYLE
Felis catus	2	SKLRALL, TTLISKI
Oryctolagus cuniculus	5	AALSSLA, KLRALLT, SKLRALL, SGPKVLM, TTAAGPP
Sus scrofa	5	SKLRALL, ASENSSA, IIAVIAL, LGGLSFL, TKFLSDV
Homo sapiens	148	ARQHSRD, ARSSSRE, AVVVSGL, DGIRGFP, DRELLLL, GKLIWKV, GLLSSIE, KAFTQGS, KKGFLCD, KLRALLT, LAGALGL, LIARKTC, LTVPPAP, MTRNLFP, NFLVSQT, NLTYVQL, QQEEGPK, RARSSSR, RSSSREN, SDKGGSF, SKLRALL, TLQVSDV, VKEQLSL, AFHQLVQ, AKTISSL, ATATAAA, DGIRGFP, DGKTLGL, EEVVQTL, ESRDETV, EVVQTLA, FQLQDGK, GGNSNTG, GKLIWKV, GSVSSAF, HASHTPG, LNRVCAE, LPPVQLP, LPTAPPE, NLTYVQL, PGPELSG,

 © 2021. The Author(s).

Supplementary Table S5 (*Continued*)

Organism	N°	Shared heptapeptides
		PGSVSSA, PLVRVNR, PSLYEES, RGGNSNT, RPVPPSP, SAIRGKI, SFDSTTA, TAAATEA, TAATTQN, VNVISGP, VRVRREL, AGPPKAE, ASENSSA, DGIRGFP, GKLIWKV, GRRTRRE, IIAVIAL, ITNTIAG, LICGLRQ, LTTKPGP, NLTYVQL, RRTRREA, SASSGKL, TLQVSDV, TTAAGPP, AEGEGAG, EFKKLPE, EGAGALL, EGEGAGA, ILNNSAS, ILTALQL, KLIERLT, LCLTNFL, LPFFTLS, LTGLLSL, NFDRSRH, PSLEKVL, QIVELLV, QSFLSDS, QTQDEIS, SIESEIV, SSTGSST, TGSSTNN, VKLIERL, VLKVFLS, VLWEIES, AAGDRGL, AARLFLR, DLTRYRE, DPVTSGL, ELASRLT, GKPFVSV, LFLRTSI, LKTYLRM, PLKGGLN, PSAALRE, SDLLIKP, SRTEDKI, SSGETRS, TAIDFVL, AARVAAS, AKVTSAC, AQKIREF, ARVAASL, ATVLKAL, DYYNPPH, EPNVLGY, ESLLHQA, FKEAVQG, GEHATVR, KILS-SIQ, LEMTPQQ, LEPLCLA, LGGLSFL, LKALSGN, LLKALSG, LLSILGS, LTLENRD, LTLGKPL, PVLLKAL, SLKEKEL, TDLEKYN, TKFLSDV, TLCEALL, VFKEAVQ, VLGYNPP, YLLSILG, AEKQLQQ, ARLLNLS, DDDDDIP, EAEKQLQ, ESDDEEQ, FDAVLYY, GAVKYLE, GVKRLEE, KKELPQD, LESD-DEE, LNLSGVN, PSGSTSP, PSLTESD, QLREAAT, RTPTVAP, SRELDHL, TEAEKQL, TSSLPPL, VKRLEEL, VYRDHSE
Mus musculus	140	ARSSSRE, AVVVSGL, EPLTVPP, GKLIWKV, KKGFLCD, LAGALGL, LESLTDR, LQLNETI, MTRNLFP, NSSLAIM, NSTIESP, QVSDVDK, RARSSSR, RSSSREN, SDKGGSF, SKLRALL, VNATEDP, AFHQLVQ, ASLATVV, ASLPAVI, ATAAATE, ATATAAA, DAAPPVI, EEVVQTL, EVVQTLA, GGNSNTG, GKLIWKV, ISGPKVL, ITSLENG, LPPVQLP, LPTAPPE, LPTQGPT, LQLNETI, PSLYEES, RGGNSNT, RPVPPSP, SAIRGKI, SPASLPA, TAATTQN, VNATEDP, VNVISGP, VRVNRLG, AAEGIYI, AAGPPKA, AGPPKAE, ARTSSDP, ASENSSA, ATTTSPQ, EESASSG, GKLIWKV, GRRTRRE, ITN-TIAG, LQLNETI, LTTKPGP, LTTLATI, MASENSS, PARTSSD, PATTTSP, QVSDVDK, RRTRREA, RSEELSF, TTAAGPP, VNATEDP, AGALLLI, ERLTGLL, ILNNSAS, KLIERLT, LCLTNFL, LTGLLSL, PSLEKVL, QTQDEIS, SFLSDSA, SIESEIV, SSARSSE, TGSSTNN, VILTALQ, VKLIERL, VLKVFLS, AAGDRGL, AARLFLR, AQILREY, DPVTSGL, ELASRLT, GAAGDRG, GNTILRT, LFLRTSI, LKTYLRM, LLSSTPV, PLKGGLN, PSAALRE, SDLLIKP, SKIINNN, SRTEDKI, TAIDFVL, TSTLDLD, ATVLKAL, ESLLHQA, FKEAVQG, FKLRSAV, LAILTRR, LEPLCLA, LGGLSFL, LLSILGS, LQGTLAS, LTLGKPL, TKFLSDV, TLCEALL, VFKEAVQ, VLGYNPP, VLSVFPL, VQLPQSL, AEKQLQQ, AITAASL, AKV-KNEV, ARLLNLS, DEDDEDT, EAEKQLQ, EEETTEA, FDAVLYY, GAVKYLE, KKELPQD, LDEDDED, LESDDEE, LFDLDED, LLIVKTV, LLPAVSS, LSGVNNL, PAVSSGK, PEEETTE, PSGSTSP, PTVAPPA, QLREAAT, REAATEA, RNQDSDN, SENGMNA, SGSTSPR, SRELDHL, TEAEKQL, TSSLPPL, VKRLEEL
Rattus norvegicus	64	KKGFLCD, KSQLSLL, NSSLAIM, QVSDVDK, RARSSSR, SDKGGSF, SKLRALL, SKSQLSL, SSLAIME, VNATEDP, ASLPAVI, DAAPPVI, PSLYEES, RPVPPSP, SHTPGSV, SPASLPA, VNATEDP, VNVISGP, ASENSSA, GRRTRRE, LTTKPGP, MASENSS, QVSDVDK, RRTRREA, RSEELSF, VNATEDP, LTGLLSL, PSLEKVL, SFLSDSA, TGSSTNN, VLKVFLS, AAGDRGL, AARLFLR, DPVTSGL, ELASRLT, PLKGGLN, SDLLIKP, TAIDFVL, TPSGKRL, ATVLKAL, DLGQLTL, FKEAVQG, LAVPQVL, LGGLSFL, TKFLSDV, VFKEAVQ, AEKQLQQ, DEEQDRD, EAEKQLQ, GAVKYLE, ITAASLP, KKELPQD, KVKNEVN, LFDLDED, LLPAVSS, PSGSTSP, PTVAPPA, QLREAAT, RLEELLP, SENGMNA, SGSTSPR, SRELDHL, TPTVAPP, VKRLEEL
Gorilla gorilla gorilla	0	–
Macaca mulatta	0	–
Pan troglodytes	3	TLQVSDV, PLVRVNR, SDLLIKP
Pteropus alecto	0	–

Supplementary Table S6 HIV-1 proteome, 3134 aa (NCBI: TaxId 11676)

Organism	N°	Shared heptapeptides
Bos taurus	14	EALLDTG, GVYYDPS, LGRIVSP, TKIEELR, AEPAADG, AVGIGAL, EQELLEL, ALDKIEE, AVFIHNF, GGIGGYS, GIGGYSA, PEPTAPP, QVTNPAT, RRNRRRR
Canis lupus familiaris	3	DGVGAVS, EAQEEEE, KIKALVE
Felis catus	0	–
Oryctolagus cuniculus	0	–
Sus scrofa	2	IQKLVGK, KIKALVE
Homo sapiens	78	AELELAE, ALDKIEE, ALFLGFL, ALGPGAT, ALVVAII, ASQIYAG, ATIMIQK, DRSIRLV, EAQEEEE, EEAELEL, EEEEVGF, ELAENRE, ELELAEN, ELRQHLL, ERAEDSG, EVVPLTE, FRDYVDR, GAITSSN, GGLVGLR, GLHTGER, GLVGLRI, GQLKEAL, IRLVNGS, ISERILS, KDLIAEI, KEATTTL, KNCSFNI, LALAALI, LASLRSL, LDKWEKI, LEELKSE, LELAENR, LGRIVSP, LLLNGSL, LPSVRKL, LQYLALA,

(*Continued*)

Supplementary Table S6 (*Continued*)

Organism	N°	Shared heptapeptides
		LRAEQAS, LRSLFGS, LTVQARQ, LVNGSLA, LWKGEGA, MTKILEP, NGTGPCT, NNSQVSQ, NWRSELY, PEPTAPP, PEREVLE, PFRDYVD, PQVPLRP, PVHAGPI, QELLELD, QGKAREF, QLIKKEK, QLKEALL, QLPPLER, REQFGNN, RGDPTGP, RKKRRQR, RQGPKEP, RRAEPAA, RRNRRRR, SGNESEG, SLRSLFG, SMTKILE, SVTVLDV, TAYFLLK, TLRAEQA, TQLLLNG, TSLIHSL, TSVITQA, VEINCTR, VELLGRR, VIEVLQA, VNGSLAL, VPLQLPP, VVPLTEE, WLEAQEE, YLALAAL
Mus musculus	76	AENREIL, AFPQGKA, AGTTSTL, ALGPGAT, ALVVAII, ARQLLSD, ARVLAEA, ATIMIQK, AVGIGAL, CNSTQLF, EAQEEEE, EEEEVGF, EEEGGER, EEGGERG, EELRQHL, ERAEDSG, FNISTSI, FRDYVDR, GAITSSN, GGLVGLR, GLHTGER, GLVGLRI, GVGAVSR, IRLVNGS, KDLIAEI, KEATTTL, KQKKSVT, KSKKKAQ, KTILKAL, KVEEANK, LAFPQGK, LALAALI, LDTGADD, LEELKSE, LEKHGAI, LFCASDA, LGILMIC, LGRIVSP, LLEELKS, LLLNGSL, LQAIHLA, LQKQITK, LQSRPEP, LSHFLKE, LVNGSLA, LWKGEGA, NGTGPCT, NTNSSSG, PEPTAPP, PFRDYVD, PLERLTL, PLTEEKI, PPLPSVR, PQVPLRP, PRGPDRP, PTGPKEM, QELLELD, QLPPLER, REQFGNN, RKKRRQR, RYPLTFG, SPLSFQT, SVLSGGE, TEVVPLT, TLELLEE, TQLLLNG, TRRELQV, TSTTVKA, TSVITQA, VIEVLQA, VLVGPTP, VNGSLAL, VPLQLPP, WLEAQEE, YELHPDK, YLALAAL
Rattus norvegicus	36	AIVALVV, ALVVAII, ATIMIQK, FRDYVDR, GPGATLE, IEEEQNK, KIEEEQN, KTILKAL, LQKQITK, LQSRPEP, PEPTAPP, PFRDYVD, RRNRRRR, TLELLEE, ARQLLSD, DLEKHGA, EAQEEEE, EEEEVGF, EEEGGER, EEGGERG, EQELLEL, FNISTSI, GGLVGLR, GVGAVSR, KVEEANK, LGILMIC, LLLNGSL, NGTGPCT, PQVPLRP, VIEVLQA, AFPQGKA, KDLIAEI, LAFPQGK, RKKRRQR, TRRELQV, YLALAAL
Gorilla gorilla gorilla	0	–
Macaca mulatta	1	VEINCTR
Pan troglodytes	1	YLALAAL
Pteropus alecto	0	–

 © 2021. The Author(s).
